# Root ethylene signalling is involved in *Miscanthus sinensis* growth promotion by the bacterial endophyte *Herbaspirillum frisingense* GSF30^T^

**DOI:** 10.1093/jxb/ert276

**Published:** 2013-09-16

**Authors:** Daniel Straub, Huaiyu Yang, Yan Liu, Tatsiana Tsap, Uwe Ludewig

**Affiliations:** Institut für Kulturpflanzenwissenschaften, Ernährungsphysiologie der Kulturpflanzen (340h), Universität Hohenheim, Fruwirthstrasse 20, D-70593 Stuttgart, Germany

**Keywords:** Biomass, diazotroph, endophyte, ethylene, *Miscanthus*, plant-growth-promoting bacteria.

## Abstract

The bacterial endophyte *Herbaspirillum frisingense* GSF30^T^ is a colonizer of several grasses grown in temperate climates, including the highly nitrogen-efficient perennial energy grass *Miscanthus.* Inoculation of *Miscanthus sinensis* seedlings with *H. frisingense* promoted root and shoot growth but had only a minor impact on nutrient concentrations. The bacterium affected the root architecture and increased fine-root structures. Although *H. frisingense* has the genetic requirements to fix nitrogen, only minor changes in nitrogen concentrations were observed. *Herbaspirillum* agglomerates were identified primarily in the root apoplast but also in the shoots. The short-term (3h) and long-term (3 weeks) transcriptomic responses of the plant to bacterial inoculation revealed that *H. frisingense* induced rapid changes in plant hormone signalling, most prominent in jasmonate signalling. Ethylene signalling pathways were also affected and persisted after 3 weeks in the root. Growth stimulation of the root by the ethylene precursor 1-aminocyclopropane 1-carboxylic acid was dose dependent and was affected by *H. frisingense* inoculation. Minor changes in the proteome were identified after 3 weeks. This study suggests that *H. frisingense* improves plant growth by modulating plant hormone signalling pathways and provides a framework to understand the beneficial effects of diazotrophic plant-growth-promoting bacteria, such as *H. frisingense*, on the biomass grass *Miscanthus*.

## Introduction

Many gramineous species maintain a close association with endophytic bacteria that are beneficial for plant growth and health (Reinhold-Hurek and Hurek, 1998). Diazotrophic, non-legume plant-growth-promoting bacteria are able to support plant growth at low nitrogen conditions by a combination of nitrogen fixation, increasing the availability of soil nutrients, promoting root growth by hormonal signalling, and controlling disease symptoms (Spaepen *et al.*, 2009). In Brazilian sugarcane, diazotrophic bacteria substantially contribute between 25% and 60% to the nitrogen acquisition of plants, but the exact value is often vague (Boddey *et al.*, 2001; Sevilla *et al.*, 2001; Taule *et al.*, 2012; Urquiaga *et al.*, 2012). Evidence for substantial non-legume nitrogen fixation in fields of temperate climates is poor and variable (Bhattacharjee *et al.*, 2008), and a better molecular understanding of the complex plant–bacterial interaction may help to increase nitrogen fixation rates.

The C4-fibre plant *Miscanthus* is a highly nutrient-efficient biomass plant and is one of the favourites for sustainable biomass production (Karp and Shield, 2008). It has been associated with the endophytic bacterium *Herbaspirillum frisingense* in the temperate climate of southern Germany. *Herbaspirillum frisingense* was isolated using nitrogen-free semi-solid medium (Kirchhof *et al.*, 2001). Genome sequencing of *H. frisingense* showed that the bacterium has all the genomic requirements to fix nitrogen and lacks several factors that may contribute pathogenic characteristics that are found in other *Herbaspirillum* strains (Straub *et al.*, 2013*a*). Many *Herbaspirillum* isolates lack nitrogen-fixation genes, including isolates closely related to *H. frisingense* (Straub *et al.*, 2013*a*). The ability to fix nitrogen of some diazotrophic *Herbaspirillum* strains has been proven in association with wild rice but not with cultivated rice (Elbeltagy *et al.*, 2001). Many *Herbaspirillum* spp. isolates have been obtained from tropical and subtropical conditions, with the nitrogen-fixing *Herbaspirillum seropedicae* investigated in most detail (Pedrosa *et al.*, 2011). However, many *H. seropedicae* isolates are also potential pathogens on various hosts, which reduces their potential versatility as biofertilizers. By contrast, the genome structure (with the lack of many systems potentially involved in pathogenicity), the colonizing characteristics, and the production of plant-growth-promoting factors suggest that *H. frisingense* might be used as a potential biofertilizer for several C4 grasses (Monteiro *et al.*, 2012*b*).

The microaerobic diazotroph *H. frisingense* invades the intercellular spaces of *Miscanthus* and barley roots without apparent damage to the host (Rothballer *et al.*, 2008). The potential nitrogen-fixing activity of this bacterial species was demonstrated using a acetylene reduction assay, PCR detection of *nif* genes (Rothballer *et al.*, 2008), and genome sequencing (Straub *et al.*, 2013*a*). *Herbaspirillum* spp. strains differ in their capacity to synthesize the plant hormone auxin and other metabolites that regulate plant growth, such as *N*-acylhomoserine lactones (Schuhegger *et al.*, 2006; von Rad *et al.*, 2008). The ethylene precursor 1-aminocyclopropane-1-carboxylate (ACC) can be used as a nitrogen source, suggesting that a biosynthetic pathway for its production or catabolism exists (Glick *et al.*, 2007; Rothballer *et al.*, 2008). Genome sequencing of *Herbaspirillum* strains have shown that ACC degradation may be a common strategy of members of the genus *Herbaspirillum* to affect plant growth (Straub *et al.*, 2013*a*).

The molecular mechanisms of how plant-associated and/or growth-promoting *Herbaspirillum* bacteria suppress the plant immune system and specifically invade the host are still unclear (Pedrosa *et al.*, 2011; Monteiro *et al.*, 2012*a*). The large variety of plant-growth-promoting bacteria and the high diversity in their genomic composition suggests that both common and strain-specific unique strategies for this interaction exist. However, the mechanisms of these potentially beneficial associations are still poorly understood.

*Miscanthus* belongs to the family Gramineae and its stem height reaches up to 4 m in one growth season. In particular, the variety *Miscanthus*×*giganteus*, a naturally occurring sterile hybrid of *Miscanthus sinensis* and *Miscanthus sacchariflorus* (Greef and Deuter, 1993) combines low nutrient, especially very low nitrogen, requirements with good agronomic properties and high biomass yields. In Germany, yields of 20–30 t ha^–1^ year^–1^ (Lewandowski and Kicherer, 1997) demonstrate the suitability of this grass as a renewable resource for energy production with an even CO_2_ balance. Many studies have confirmed that the use of a mineral fertilizer has little or no significant effect on *Miscanthus*×*giganteus* biomass production (Himken *et al.*, 1997; Christian *et al.*, 2008). According to Christian *et al.* (1997), only 19% of the total plant nitrogen was derived from the introduced fertilizer when nitrogen distribution and balance was examined after the application of an ^15^N-labelled chemical fertilizer (Christian *et al.*, 1997). Model calculations on real biomass-yield data suggest a profound nitrogen input by nitrogen-fixing bacteria in the seasonal nitrogen balance (Davis *et al.*, 2010). In addition to *Herbaspirillum*, alpha-, gamma- and deltaproteobacteria have been found associated with the leaves of *Miscanthus* (Straub *et al.*, 2013*b*).

In this study, the growth of young *Miscanthus* seedlings was found to benefit from inoculation with the betaproteobacterium *H. frisingense.* The growth-promoting potential of this bacterium was dependent on the nitrogen supply. *H. frisingense* affected the signalling of plant hormones, namely ethylene signalling, in root growth. Although nutrient concentrations in treated seedlings were not affected, the improved root growth resulted in improved overall nutrient acquisition and biomass production. These results identify plant-growth-promoting bacteria functions of this diazotrophic endophyte, which may be beneficial for sustainable biomass production with *Miscanthus*, especially for establishment from seeds and maintenance in marginal soils.

## Material and methods

### M. sinensis growth

Surface-sterilized *M. sinensis* seeds (10min in 70% ethanol, rinsed with sterile water, and dried) were germinated in quartz sand (0.3–0.8mm diameter), which was washed with HCl (rinsed with tap water, pH <1 adjusted with HCl, incubated overnight, rinsed with deionized water to pH >5) to wash out trace nutrients, biological contaminations, and dust. Prior to sowing, the sand was fertilized with modified Hoagland solution [1mM KH_2_PO_4_, 0.5mM MgSO_4_, 50 µM Na_2_EDTA, 50 µM FeSO_4_, 9 µM MnCl_2_, 0.765 µM ZnSO_4_, 0.32 µM CuSO_4_, 16nM (NH_4_)_6_Mo_7_O_24_, 46 µM H_3_BO_3_, 1mM CaCl_2_] containing 1mM ammonium nitrate. After 2 weeks, plants were transferred individually to pots (9cm diameter) with HCl-washed quartz sand and incubated under conditions of 14h light, 24 °C/10h dark, 19 °C. The pots were watered three times a week: twice with modified Hoagland solution without any nitrogen source and once a week with modified Hoagland solution containing 50 µM (low nitrogen) or 250 µM (higher nitrogen) ammonium nitrate. In some experiments, the nitrogen was supplied in a 10% ^15^N-enriched form. For ethylene growth tests, the plants were watered as above once a week with nutrient solution containing 50 µM ammonium nitrate, and twice a week with 0, 0.3, 1, 5, and 20 µM ACC (Sigma-Aldrich).

Three-week-old plants were inoculated with *H. frisingense* GSF30^T^ (Straub *et al.*, 2013*a*) The bacteria were grown for 2 d in liquid Luria–Bertani medium supplemented with 50mg l^–1^ of kanamycin and harvested by centrifugation for 5min at 4000rpm at 4 °C. The pellet was washed with water and resuspended in watering solution to a final OD_600_ of ~0.1). Each pot was watered with 40ml of solution containing bacteria at about 10^9^ c.f.u. ml^–1^. For control plants, the bacterial solution was autoclaved. This inoculation step was repeated after a further 3 d. After 3 weeks, the plants were harvested. The sand was washed away with tap water, and the root was dried briefly with paper and weighed for fresh biomass determination. The significance was analysed using SAS System software Release 8.01, using one-way or two-way analysis of variance (ANOVA) with values of *P* as indicated.

### Root morphology

Roots were excised from the sand culture, scanned, and analysed with a WinRHIZO system (Regent Instruments, Canada) for the number and length of roots of different diameter.

### Element analysis

Roots and shoots were harvested separately, washed, dried for 2 d at 60 °C, and ground to a fine powder. The concentration of total nitrogen was determined using a EuroVector elemental analyser (HEKAtech, Wegberg, Germany). For the other elements, 10–18mg of tissue was dissolved in 65% HNO_3_ and heated in four increasing steps up to a maximal temperature of 220 °C at 160 bar in a microwave (UltraClave3; MLS, Germany). Elements were determined by inductively coupled plasma/optical emission spectrometry (PS1000; Leeman Labs, Lowell, MA, USA).

### Sequencing

Plants were harvested at 1 p.m. and immediately frozen in liquid nitrogen and stored at –80 °C. The mRNA was isolated from 100mg of plant material with an RNeasy Plant Mini kit (Qiagen) and transcribed to cDNA, which was sequenced using Illumina technology at GATC Biotech AG (Germany). *De novo* assembly and mapping was also done at GATC Biotech AG.

### Differentially expressed genes

Analysis of differentially expressed genes used a method similar to that described previously (Straub *et al.*, 2013*b*). Fold changes and relative read numbers were determined by DESeq version 1.8.3 (Anders and Huber, 2010) using Bioconductor. The procedure followed the instructions of the manufacturer (Analysing RNA-Seq data with the DESeq package; Working without any replicates: http://www.bioconductor.org/help/course-materials/2011/BioC2011/LabStuff/DESeq.pdf), and input were raw count tables. Contigs were filtered for meaningful expression by deleting contigs with the lowest DESeq base mean. The remaining 27.3% contigs represented more than 95% of reads.

### Transcript annotation

Contigs were annotated with Mercator (http://mapman.gabipd.org/web/guest/mercator) and classified according to MapMan (http://mapman.gabipd.org/web/guest/mapman) functional plant categories (bins). Therefore, SwissProt/UniProt Plant Proteins (PPAP), TIGR5 rice proteins, clusters of orthologous eukaryotic genes database (KOG), conserved domain database (CDD) and Interpro scans were enabled, allowing multiple bin assignments. ‘Unassigned’ bins were considered with equal weight when assigning bin codes. Metabolic pathways were visualized with MapMan 3.5.1R2 and its built-in module PageMan, showing DESeq’s fold change values.

### Real-time PCR

Plants were harvested at 1 p.m., immediately frozen in liquid nitrogen and stored at –80 °C. RNA was extracted from 100mg of fine-milled plant material with an innuPREP Plant RNA kit (Analytik Jena). Contaminating genomic DNA was removed with an RNase-Free DNase Set (Qiagen), according to the manufacturer’s instructions. High-quality RNA was chosen based on photometric measurements (NanoDrop 2000c; Thermo Scientific) and formaldehyde–agarose gel electrophoresis according to the RNeasy Mini Handbook, Appendix C (Qiagen) and was immediately transcribed to cDNA or frozen at –80 °C. SsoFast EvaGreen Supermix reaction cocktail (Bio-Rad) was used with a C1000TM thermal cycler chassis and a CFX384 Real-Time PCR Detection System (Bio-Rad) and evaluated with CFX Manager 2.1 software (Bio-Rad). Primers were selected with Primer3Plus (Untergasser *et al.*, 2012). Primer sequences and expected product lengths of the selected reference genes (*PP2AA2*, *Actin-1*, and *DnaJ*; see Supplementary Materials and Methods at *JXB* online) and other genes can be found in Supplementary Table S1 in Supplementary at *JXB* online. Melting-curve analysis and agarose gel electrophoresis confirmed the specificity and quality of the PCR products.

### Protein isolation and 2D separation of *Miscanthus* proteins

Total proteins were isolated from approximately 2.5g of frozen whole plants per biological replicate via acetone precipitation (Liu *et al.*, 2006). Isoelectric focusing of proteins was performed with 1000 µg of protein extract using an IPGphor3 isoelectric focusing unit (GE Healthcare) and 24cm immobilized, non-linear pH 3–11 gradients (Imobiline Dry Strips; Amersham Biosciences). Rehydration was performed at 20V overnight. The voltage settings for the isoelectric focusing were 100V for 4h, 300V for 2.5h, 300–1000V gradient for 8h, 1000–8000V gradient for 8h, 8000V for 7h, and 8000–50V gradient to a final setting of 87 900 Vhs. Equilibration of the dry strips was performed as described previously (Sauer *et al.*, 2006). Proteins in the equilibrated dry strips were then separated on the basis of their molecular weight by 12% SDS-PAGE using the Ettan DALTsix Electrophoresis System (GE Healthcare).

After electrophoresis, the proteins were stained with a modified colloidal Coomassie blue stain (Sauer *et al.*, 2006) for 72h on an orbital shaker. Stained gels were imaged using a Typhoon Trio+ Imaging System (GE Healthcare). The resulting gel image files were exported to Progenesis SameSpots (Non-linear Dynamics, http://www.nonlinear.com). Proteins were accepted as differentially accumulated when they displayed a fold change of >1.8 and were significant in Student’s *t*-test at a significance level of *P* <0.05.

### Microscopy

A green fluorescent protein (GFP)-tagged *H. frisingense* GSF30^T^ strain was observed by confocal microscopy (Leica DMRE microscope equipped with a confocal head TCS SP; Leica, Wetzlar, Germany).

## Results

### Growth promotion and colonization of M. sinensis with H. frisingense

**Fig. 1.**
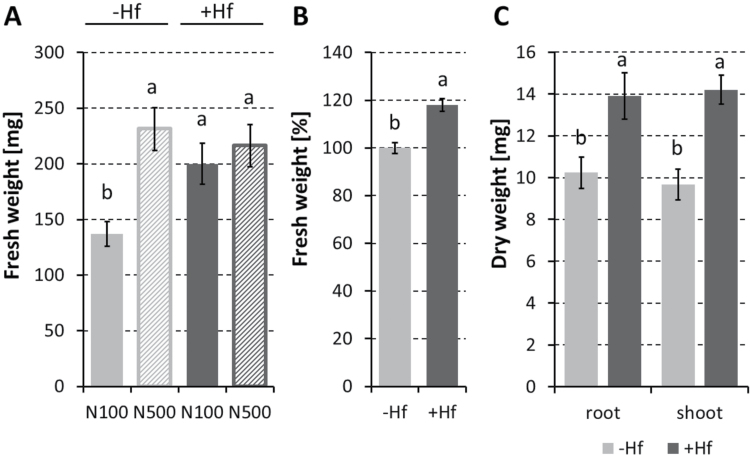
Growth promotion of *M. sinensis* by *H. frisingense* GSF30^T^ (Hf). The graphs show total biomass from 6-week-old plants. (A) Fresh weight [mg, ±standard error (SE)] of seedlings grown with (+Hf, dark grey bars) or without *H. frisingense* (–Hf, pale grey bars) under low (N100, 50 µM NH_4_NO_3_; 12 plants) or higher (N500, 250 µM NH_4_NO_3_; 13 plants) nitrogen levels. Different lower-case letters indicate a significant difference by ANOVA test (*P* < 0.05). (B) Fresh weight (% compared with –Hf, ±SE) following *H. frisingense* inoculation (*n*=11 independent experiments; total 223 plants +Hf and 259 plants –Hf) under N100 conditions. Different lower-case letters indicate a significant difference by ANOVA test (*P* < 0.001). (C) Dry weight (mg, ±SE) of 6-week-old seedlings, separated for roots and shoots, without or with *H. frisingense* inoculation under N100 conditions.

Seedlings of *M. sinensis* were grown in sand and watered with nutrient solutions that contained different concentrations of nitrogen. In low nitrogen conditions (100 µM total nitrogen watered once a week, N100), *M. sinensis* accumulated less biomass than in 500 µM nitrogen (N500), suggesting that the nitrogen supply limited the plant growth at low nitrogen (Fig. 1A). After 3 weeks of inoculation with *H. frisingense* GSF30^T^, the total fresh biomass of inoculated plants showed a significant increase to the same level as control plants fertilized with the higher N500 dosage. By contrast, the growth-promoting effect of *H. frisingense* GSF30^T^ was absent when the plants received the higher nitrogen supply (Fig. 1A).

Although some variability in the plant-growth-promoting effect in individual experiments was apparent over a period of 4 years, growth promotion overall in more than 12 independent experiments revealed a significant increase of about 20% higher total fresh biomass (Fig. 1B, *P* < 0.001). In a typical successful growth-promoting experiment, the dry biomass of both roots and shoots increased by almost 40% (Fig. 1C, *P* < 0.01).

The successful colonization of roots and shoots of *Miscanthus* seedlings by *H. frisingense* GSF30^T^, or absence of the bacteria in seedlings grown from surface-sterilized seeds, was monitored using plant- and bacteria-specific primer pairs. The bacterial DNA was never detected from plants grown in the absence of the bacteria, but was always detected in those that showed beneficial growth effects following colonization by *H. frisingense* GSF30^T^ (Supplementary Fig. S1 at *JXB* online).

**Fig. 2.**
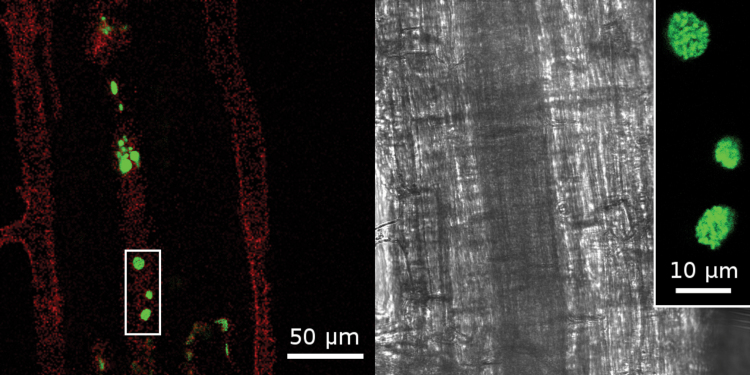
Green fluorescent spots in *M. sinensis* roots inoculated with GFP-labelled *H. frisingense*. Bacterial aggregates were visible in the intercellular apoplastic space. Bright field image (right) and fluorescence image (left). The red colour indicates residual background fluorescence from the cell wall. The inset shows a magnification of the bacterial colony aggregates. (This figure is available in colour at *JXB* online.)

The localization of the growth-promoting bacteria in young seedlings was investigated using a GFP-labelled *H. frisingense* strain under confocal microscopy. The fluorescence of bacteria was detected in the plant apoplast, where aggregates of fluorescent spots were detected, both in roots (Fig. 2) and shoots (Supplementary Fig. S2 at *JXB* online). This spot-like aggregation differred from the colonization pattern of barley (Rothballer *et al.*, 2008), where a stronger colonization and a more uniform apoplastic pattern has been observed.

### Effect on nutrient uptake and root morphology

**Fig. 3.**
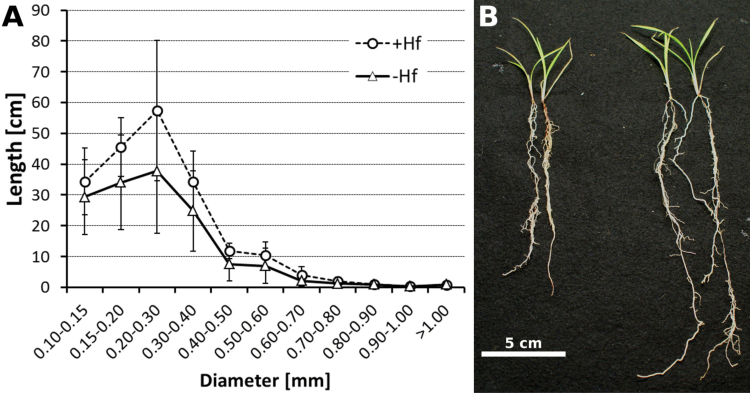
Root morphology and fine root structural changes of *M. sinensis* following inoculation with *H. frisingense*. (A) Root length of different diameter classes without inoculation (triangles) and with inoculation (circles). Quantitative root parameters were deduced using a WinRHIZO system. (B) Seedling appearance without inoculation (left) and with inoculation (right). Note the increased root system. (This figure is available in colour at *JXB* online.)

**Fig. 4.**
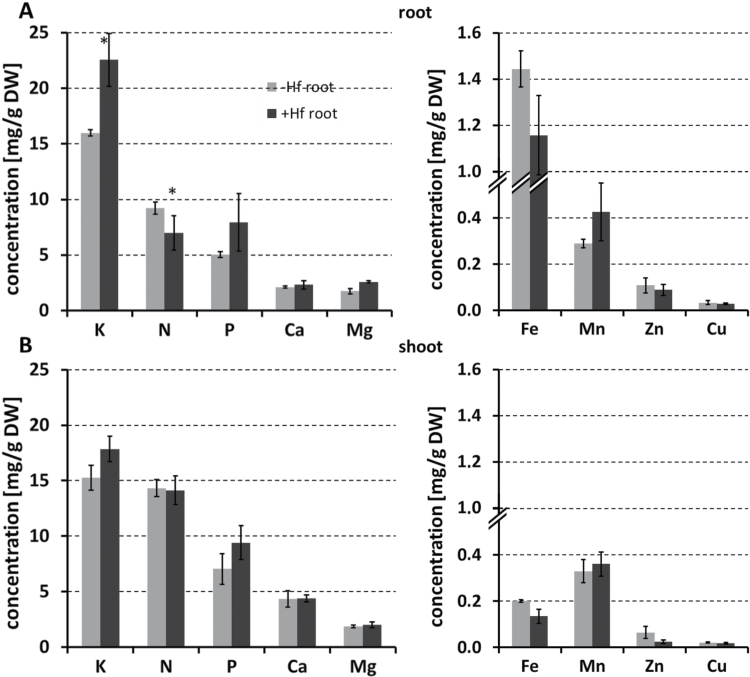
Differential element concentrations in roots (A) and shoots (B) without (grey bars) and with (black bars) *H. frisingense* inoculation. Macroelements (left graphs) and micronutrients (right graphs) are given in concentrations [mg/g dry weight (DW)]. Asterisks indicate significant differences at the *P* < 0.05 level (one-way ANOVA).

The beneficial effect of *H. frisingense* inoculation on root morphology was quantified from washed roots. These were scanned, and the total root length and number of laterals and fine roots were determined and analysed. Compared with controls that received autoclaved bacteria, inoculated seedlings showed a clear tendency towards an increase in fine-root structures, laterals, and in the total root length (Fig. 3), yielding a larger root system (Fig. 3B). Despite the larger surface area to absorb nutrients, the concentrations of macro- and micronutrients only showed marginal differences in potassium and nitrogen in roots (Fig. 4). While potassium was higher in roots in the presence of *H. frisingense*, a reduced nitrogen concentration was detected in roots upon inoculation. In the shoot, the nutrient concentrations were not affected by the diazotrophic *H. frisingense.* Due to the higher biomass in roots and shoots, however, the total content of all nutrients was increased, but this increase was only significant for K, P, Mg, and Mn in the shoot (Supplementary Fig. S3 at *JXB* online). When ^15^N-enriched nitrogen (10%) was applied for 3 weeks, the relative ^15^N/^14^N ratios in the tissue did not differ in non-inoculated and inoculated plants. Although a minor contribution of nitrogen via nitrogen fixation cannot rigorously be excluded by these experiments, a large contribution by nitrogen fixation appears unlikely, as a dilution effect of the ^15^N/^14^N ratio by potential atmospheric nitrogen fixation was not observed (Supplementary Fig. S4 at *JXB* online).

### Transcriptome changes following inoculation

**Fig. 5.**
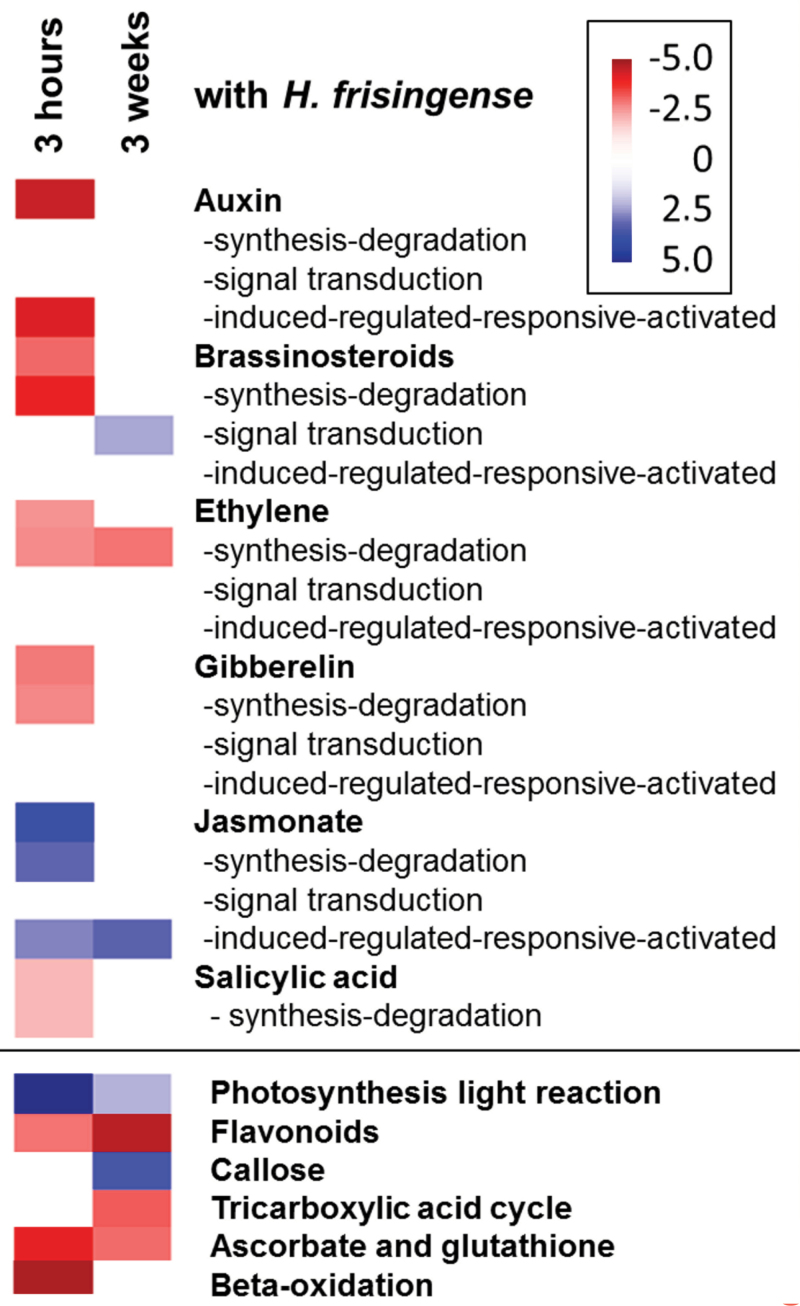
Differential expression of *M. sinensis* transcript categories related to hormones and key metabolic functions in response to *H. frisingense* inoculation. Red indicates lower expression of gene categories compared with non-inoculated plants, while blue indicates higher expression. Non-significant differences (*z*-score <1.96) are shown in white. Colouring is according to the *z*-scores of the bin-wise Wilcoxon test. A *z*-score of ±1.96 represents a *P* value of 0.05. The plot was generated using PageMan.

The early (3h) and long-term (3 weeks) changes in gene expression by *H. frisingense* inoculation were investigated with quantitative mRNA sequencing (RNA-Seq). The early response of *M. sinensis* to the inoculation with *H. frisingense* involved a consistent, prominent upregulation of genes involved in jasmonate signalling and biosynthesis, and in response genes (Fig. 5). Genes involved in the biosynthesis and signalling of other plant hormones were also affected in a concerted manner, but the individual gene expression changes were mostly only marginal. Despite this, absciscic acid, auxin, brassinosteroids, and gibberellin signalling appeared significantly lower at 3h after inoculation with *H. frisingense* (Fig. 5). However, the altered signalling apparently only persisted after 3 weeks for jasmonate and ethylene, whereas the expression changes appeared more variable for brassinosteroid signalling.

An overview of changes in various metabolic pathways 3h after inoculation showed a highly significant (*P*<0.00001) upregulation of photosynthesis-related genes and a downregulation of β-oxidation genes (Fig. 5 and Supplementary Fig. S5 at *JXB* online). Genes involved in terpene/flavonoid synthesis and redox metabolism involving ascorbate and glutathione were also collectively repressed, and these gene expression differences persisted after 3 weeks. By contrast, callose metabolism only appeared to be upregulated after 3 weeks, whereas genes involved in the tricarboxylic acid cycle were repressed after 3 weeks post-inoculation (*P*<0.003; Fig. 5 and Supplementary Fig. S6 at *JXB* online). Less prominent differences in gene expression categories indicated that several different metabolic traits were affected by inoculation, but, except for the jasmonate response, there were surprisingly few defence genes activated, which may explain why *H.frisingense* can effectively invade and colonize the plant (Supplementary Fig. S7 at *JXB* online).

### Differential gene expression related to plant hormone signalling

**Fig. 6.**
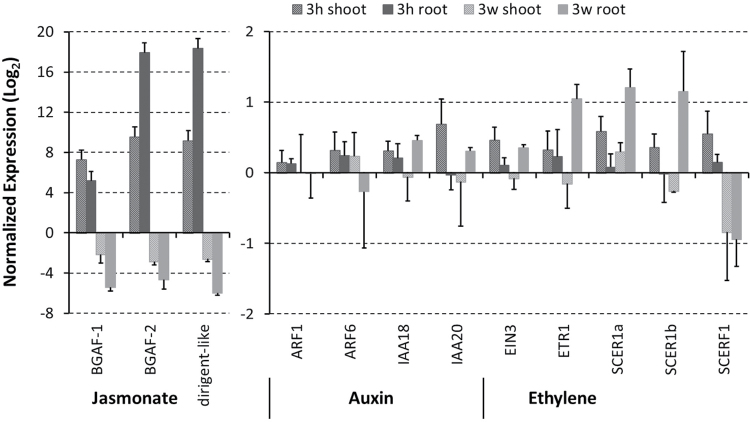
Normalized expression of *M. sinensis* transcripts related to hormones in response to *H. frisingense* (qRT-PCR, log_2_ scale). Note the different scale of jasmonate-regulated genes (*BGAF-1*, *BGAF-2*, and *dirigent-like*; left graph). Auxin-responsive genes were: auxin response factors *ARF1* and *ARF6*, and the auxin-responsive *IAA18* and *IAA20* genes. Ethylene-related genes were ethylene insensitive 3 (*EIN3*), ethylene-resistant 1 (*ETR1*), the sugarcane ethylene receptor-like *SCER1-like SCER1a* and *SCER1b*, and the ethylene response factor *SCERF1-like.* A *SCERF2*-*like* gene (Cavalcante *et al.*, 2007) was not identified in *Miscanthus*. 3h, 3h after inoculation; 3 w, 3 weeks after inoculation. Error bars: +SE of three (3h) or two (3 w) biological replicates. Qualitatively similar results were obtained from two further replicates.

The potential involvement of jasmonate, auxin, and ethylene signalling in the rapid and long-term plant response to the bacterial inoculation was evaluated using quantitative real-time PCR (qRT-PCR) of selected marker genes. Marker genes responding to jasmonate in various plant species include two β-glucosidase aggregating factor genes (*BGAF-1* and *BGAF-2*) and a dirigent-like protein gene. The expression of these genes was highly induced in the transcriptome dataset. When analysed by qRT-PCR, the expression of these genes was increased by several orders of magnitude after 3h of inoculation, in both roots and shoots. However, these genes were repressed after 3 weeks, especially in the roots (Fig. 6).

By contrast, only very minor changes in the expression of several auxin-responsive genes, such as the moderately expressed auxin response factors *ARF1*, *ARF6*, *IAA18*, and *IAA20* was observed at early and late stages, consistent with the minor expression changes in the transcriptome data set.

Finally, the gene expression of several ethylene-related genes was monitored. This included the highly abundant ethylene receptor ethylene insensitive 3 (*EIN3*) and several low-abundant ethylene-responsive genes. These genes included the ethylene-resistant 1 gene (*ETR1*), two sugarcane ethylene receptor 1-like genes (*SCER1a* and *SCER1b*), and the sugarcane ethylene response transcription factor 1-like (*SCERF1*), which had been analysed previously in a sugarcane–*Herbaspirillum* interaction (Cavalcante *et al.*, 2007). While the expression of the ethylene receptor *EIN3* was not affected by the inoculation, *ETR1* and *SCER1*-like genes were upregulated in roots (Fig. 6). By contrast, *SCERF1-like* was repressed in roots and shoots after 3 weeks of growth with *H. frisingense*. These results suggested that the presence of the bacteria induced long-term differences in jasmonate and ethylene signalling, which were most prominent in the root. The relative changes in the qRT-PCR expression level of these marker genes correlated qualitatively, but not quantitatively, with the expression changes deduced from the transcriptomics data and varied slightly within biological replicates.

### Growth promotion of *M. sinensis* with *H. frisingense* and ethylene

The identification of prominent differences in the ethylene signalling in *Miscanthus* roots by diazotrophic growth-promoting bacteria parallels results obtained with sugarcane (Nogueira *et al.*, 2001; Cavalcante *et al.*, 2007). Whether an external supply of ethylene or its precursor 1ACC altered root growth was therefore investigated. The root biomass of *M. sinensis* showed a dose-dependent ethylene sensitivity in pot experiments. The addition of (labile) ACC to the nutrient solution increased plant biomass only at an intermediate concentration, whereas seedlings inoculated with *H. frisingense* already had an increased biomass without the addition of ACC (Supplementary Fig. S8 at *JXB* online). The application of ACC to inoculated seedlings increased the biomass to a maximum when the concentration (5 µM) was higher than that in the absence of *H. frisingense.* This effect may partially be related to ACC deaminase of *H. frisingense*, which may decrease the amount of plant-available ethylene, which in turn affects root proliferation. This bell-shaped biomass distribution of total seedlings with respect to ACC supplementation was probably due to a primary effect on the root growth, which was visibly stimulated in the seedlings as a function of the ethylene availability (Supplementary Fig. S9 at *JXB* online). The differential sensitivity to external ethylene strengthens the findings of long-term differentially regulated *ETR*/*ER* and *ERF* genes in the roots (Fig. 6) and underlines the importance of ethylene in endophyte–plant interactions. However, these data also suggested that further plant-growth-promoting actions are conferred by *H. frisingense*, which are unrelated to ethylene.

### Differential proteomics

Differentially abundant proteins (fold change >1.8, *P*<0.05) in *M. sinensis* seedlings grown without or with *H. frisingense* for 3 weeks were determined. Of the 14 protein spots identified, 12 were more abundant and two were less abundant after *Herbaspirillum* inoculation (Supplementary Fig. S10 at *JXB* online). The annotation of the differential spots is given in Table 1. Several differentially regulated proteins were related to primary metabolism, but none was related to plant hormone signalling. Three spots were annotated as fructose-bisphosphate aldolase: two of them were upregulated, whereas one was down-regulated. Two spots were close to each other, which could represent different phosphorylation states of the same protein, whereas the downregulated spot might indicate a lighter and smaller complex. Improved photosynthesis could be suggested by the higher abundance of the large subunit of ribulose-1,5-bisphosphate carboxylase oxygenase (Rubisco) and serine–glycine hydroxymethyltransferase (Table 1). Aconitate hydratase was present in four differential spots, which differed only in their pI values (Table 1). These might therefore again represent different phosphorylation states. Two of the upregulated proteins were identified as β-tubulin and TCP-1 γ-chaperonin, which are both involved in cytoskeleton formation. Tubulin is a vital component of the cytoskeleton and TCP-1 γ-chaperonin folds as a complex with various proteins, including actin and tubulin. The differentially regulated cinnamyl alcohol dehydrogenase is involved in phenolpropanoid and lignin biosynthesis, whereas ascorbate peroxidases are involved in stress metabolism.

**Table 1.** *Differentially abundant proteins of M. sinensis grown 3 weeks with H. frisingense GSF30* ^*T*^Spot number, significance level, fold changes, annotation, coverage, number of unique peptides, predicted mass, and the relative RNA expression differences (from RNA-Seq) of 14 differential spots are given. Localization of the individual spots in the 2D gels is shown in Supplementary Fig. S8

Spot	ANOVA (*P*)	Fold	Protein annotation	Best hit	% Coverage	Unique peptides	kDa	Expression
3156	0.004	4.9	β-Tubulin	gi|293331107	41	24	55	0.0
961	0.005	1.9	TCP-1 chaperonin, subunit gamma	gi|242036525	33	26	61	0.0
3096	0.002	2.9	Cinnamyl alcohol dehydrogenase	gi|242049212	21	10	44	–0.1
3068	0.000	4.6	Rubisco large subunit	gi|48478779	31	18	53	0.1
1146	0.021	1.8	Serine–glycine hydroxymethyltransferase	gi|242068375	37	21	52	–0.3
3092	0.014	–2.3	Fructose-bisphosphate aldolase	gi|242059597	19	9	39	–0.2
3102	0.007	2.4			39	20		
3139	0.030	2.1			57	23		
3074	0.033	3.3	Aconitate hydratase	gi|242037013	26	30	107	–0.1
3081	0.016	3.4			18	24		
3084	0.013	2.4			17	21		
3110	0.018	3.8			15	21		
2073	0.028	1.8	l-Ascorbate peroxidase 2	gi|226897533	23	5	27	–0.2
2194	0.004	–3.3	l-Ascorbate peroxidase 1	gi|242041317	28	9		

## Discussion

In this study, *M. sinensis* seedlings were found to profit from inoculation with the diazotrophic *H. frisingense* under conditions of a low nitrogen supply (Fig. 1). *H. frisingense* is genetically related to the sugarcane, rice, sorghum, and maize colonizer *H. seropedicae*, which, in contrast to *H. frisingense*, occasionally confers disease symptoms on some plant host genotypes (Monteiro *et al.*, 2012*b*). The absence of disease symptoms in *Miscanthus* inoculated with *H. frisingense* may be explained by the fact that *H. frisingense* lacks several genetic components that are involved in pathogenic functions (Straub *et al.*, 2013*a*). *H. frisingense* is also closely related to *Herbaspirillum* strains isolated from other habitats, such as strains that were isolated from the rhizosphere of Australian phragmites and from Japanese well water (Straub *et al.*, 2013*a*).

Among the non-legumes, the direct contribution of nitrogen fixation (25–60%) by diazotrophic endophytic communities to nitrogen nutrition is probably best verified in Brazilian sugarcane (Boddey *et al.*, 2001; Taule *et al.*, 2012; Urquiaga *et al.*, 2012). The evidence for non-legume nitrogen fixation in temperate climates from field studies is less clear; for example, the diazotroph *Azospirillum* exerts its beneficial roles on growth, not via nitrogen fixation (Bashan *et al.*, 1989). However, the extraordinary nitrogen efficiency in *Miscanthus* (Christian *et al.*, 1997, 2008) suggests that, at least in this grass, diazotrophs contribute to this nitrogen efficiency. Our results suggest that, at least in very young seedlings, the potential contribution of nitrogen fixation to nitrogen nutrition is rather small, although it could not be rigorously excluded from the above experiments that some nitrogen fixation occurred. Significant nitrogen fixation may occur at later developmental stages, and it is interesting to note that alphaproteobacterial and rhizobacterial DNA sequences have been identified in field-grown adult *Miscanthus* leaves (Straub *et al.*, 2013*b*). While the nitrogen concentration was even reduced in inoculated roots, the potassium concentration was increased. The nutrient concentrations in the shoot were not affected by the inoculation (Fig. 4). Other plant-growth-promoting rhizobacteria, including the well-studied *Azospirillum*, increase nutrient concentrations under certain conditions (Spaepen *et al.*, 2009). Whether *H. frisingense* can increase the availability of other soil nutrients, such as phosphorus, which was slightly but not significantly increased, should be analysed in future studies.

Diazotrophic bacterial grass colonizers, such as *H. seropedicae* and *Gluconacetobacter diazotrophicus*, promote root growth by manipulating plant hormone signalling. In this way, the root surface area available to absorb nutrients is increased and consequently allows the production of more total biomass. The transcriptome analysis identified that the jasmonate response and ethylene signalling were persistently altered by the presence of *H. frisingense* (Fig. 8). These plant hormones are well-known key players in abiotic and biotic stresses (Thatcher *et al.*, 2005). The prominent upregulation of jasmonate-inducible defence proteins after 2 weeks of inoculation was reported for the closely related endophyte *Azoarcus* spp. upon invasion of *Oryza sativa* cultivars (Miche *et al.*, 2006). In sugarcane, a partial overlap in the activation of resistance genes induced by methyl jasmonate and by colonization by *H. seropedicae* and *Herbaspirillum rubrisubalbicans* has been noted (Rocha *et al.*, 2007), suggesting a conserved initial jasmonate signalling response in diverse endophyte–grass host interactions. This jasmonate response occurred before the establishment of an effective association (Rocha *et al.*, 2007).

In the *Miscanthus* transcriptome and in qRT-PCR, three jasmonate-induced genes (*BGAF-1*, *BGAF-2* and *dirigent-like*) were more highly expressed by orders of magnitude after 3h (Fig. 6). Three weeks after inoculation, the qRT-PCR data suggested that these jasmonate response genes were significantly repressed. BGAF is a Poaceae-specific protein that can bind β-glucosidase, which is involved in the jasmonate-dependent defence against pathogens in young maize plants. Herbivores release toxic aglycons from hydroxamic acid glucosides, which are one of the major defence compounds in members of the family Poaceae (Niemeyer, 1988). The predominant hydroxamic acid glucoside in maize is 2,4-dihydroxy-7-methoxy-1,4-benzoxazin-3-one (DIMBOA)-Glc, whose aglycone DIMBOA has a strong inhibitory effect on *Agrobacterium tumefaciens* (Sahi *et al.*, 1990). BGAF aggregates and concentrates β-glucosidase, and protects BGAF and β-glucosidase from degradation, but has no effect on β-glucosidase activity (Esen and Blanchard, 2000). BGAF contains a jacalin-related lectin domain and a disease-response (dirigent protein) domain (Kittur *et al.*, 2007).

The rapid, strong upregulation of these jasmonate-related genes after inoculation with *H. frisingense* suggested that a common early defence response in *M. sinensis* occurs. However, after establishment of the association, the respective genes were even suppressed by *H. frisingense*, which is well in accordance with its endophytic life style. Furthermore, little indication for further defence responses (except for changes in redox metabolism) were obtained from the transcriptome and proteome data, suggesting that *H. frisingense* is able to at least partially bypass or suppress the plant defence response. The weak colonization density (Fig. 2) may contribute to this minor defence reaction.

**Fig. 7.**
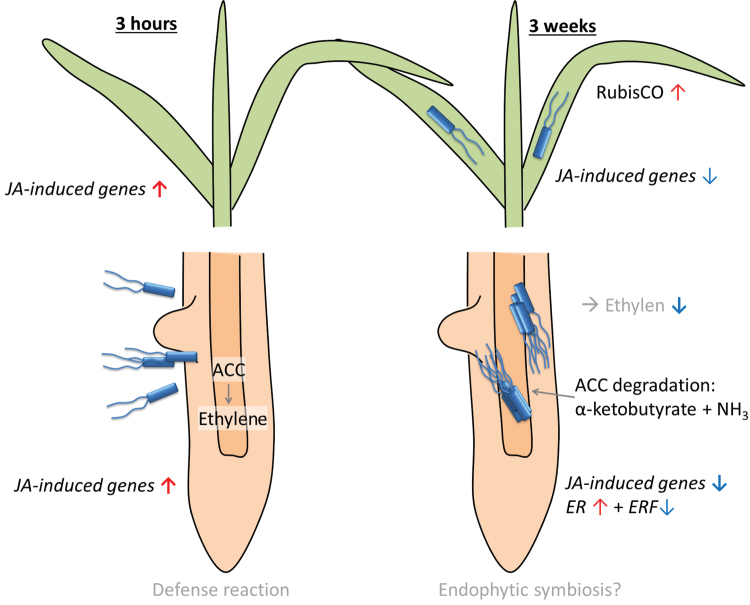
Mechanistic scheme of the interaction of *M. sinensis* and *H. frisingense*. The beneficial effect of *Herbaspirillum*-induced root hormone signalling leads to higher root biomass, improved nutrient absorption, and, as a consequence, also increases the shoot biomass. Red arrows: up-regulated genes, blue arrow: down-regulated genes. *JA*, jasmonate; *ER*, ethylene receptor*; ERF*, ethylene response factor. (This figure is available in colour at *JXB* online.)

Ethylene is an essential signalling molecule for growth and developmental processes in plants (Wu *et al.*, 2009). *H. frisingense*, as well as all other fully sequenced, plant-associated *Herbaspirillum* strains, produces the enzyme ACC deaminase (Rothballer *et al.*, 2008). This enzyme degrades the ethylene precursor ACC and locally reduces plant-available ethylene. Divergent bacterial species are able to reduce the local ethylene levels via ACC deaminase activity and hence alter the root morphology (Contesto *et al.*, 2008). Furthermore, ACC deaminase can protect plants from various stresses and in this way improve growth (Glick, 2004). In the established *Miscanthus–Herbaspirillum* interaction, ethylene receptors appeared specifically upregulated in roots, while ethylene response factors were repressed (Fig. 6). This is consistent with the assumption that *H. frisingense* can actively reduce ethylene levels in plant roots by ACC deaminase, which competes with the plant ACC oxidase for ACC (Glick, 2005). In rapeseed seedlings, the effects of ACC deaminase-containing bacteria on biomass gain is dependent on the nutritional status of the plants, and the presence of the bacteria often reduces the shoot nutrient content, which was not the case here (Belimov *et al.*, 2002). Positive effects of plant root-growth-promoting bacteria on ethlyene signalling are well established in heavy metal stress (Safronova *et al.*, 2006) and saline stress (Saravanakumar and Samiyappan, 2007), where the stress-induced root growth suppression is counteracted by ACC deaminase-containing bacteria. In sugarcane, ethylene-related genes are differentially affected by different diazotrophic bacterial strains (Nogueira *et al.*, 2001; Cavalcante *et al.*, 2007), in a similar way overall to here in *Miscanthus*. The lower availability of ethylene in *Miscanthus* roots is consistent with the upregulation of the ethylene receptor genes and repression of the response factors, but a *SCER1* ethylene receptor is induced in sugarcane after inoculation with *G. diazotrophicus* or *H. rubrisubalbicans*, while an ethylene-responsive factor is only induced by the endophyte *G. diazotrophicus* (Cavalcante *et al.*, 2007), which does not contain ACC deaminase to degrade the ethylene precursor (Straub *et al.*, 2013*a*). This persistent regulation of ethylene-responsive genes (including up- and downregulated genes) is similar in rice roots 7 d after inoculation with *H. seropedicae* (Brusamarello-Santos *et al.*, 2012). However, besides this common regulation of jasmonate- and ethylene-responsive signalling (Fig. 7), only very little overall overlap in the endophyte-induced transcriptiome profile is seen with other grasses and *Miscanthus.* This may indicate endophyte strain-specific and plant species-specific responses during interaction with diverse bacterial species. Furthermore, there is also little overlap with grass responses to fungal endophytes, except for the differential regulation of genes involved in redox metabolism, although both endophytes colonize and feed from the apoplast (Tanaka *et al.*, 2012).

*H. frisingense* (as well as *H. seropedicae*) produces the plant growth-promoting factor indoleacetic acid (IAA) (Rothballer *et al.*, 2008). *IAA* and *ARF*-like genes encode transcription factors that are expressed in a tissue-specific manner and respond to light and/or auxin (Cheong *et al.*, 2002; Jain and Khurana, 2009; Brusamarello-Santos *et al.*, 2012). A collective minor repression of *IAA* and *ARF* genes was observed in rice roots 7 d after inoculation with *H. seropedicae*, but there were genotype differences (cv. Cateto zebu and Nipponbare). Here, a differential expression of specific auxin-responsive genes was marginal and not persistent; the individual expression level differences were negligible (Fig. 6), suggesting only a minor change in the auxin signalling in the beneficial association. The cross-talk of auxin signalling and ethylene has also been studied with ACC deaminase-containing and -deficient bacteria in rapeseed. It was suggested that auxin, which activates ACC synthase in plants, must be balanced with ethylene to maintain optimal auxin signal transduction in the root (Stearns *et al.*, 2012).

Few metabolic categories differed following inoculation, and this included stimulation of primary metabolism and repression of some secondary metabolism pathways (Fig. 5). Differences in the primary metabolism were suggested by the differential proteomes after 3 weeks (Table 1), which is consistent with the higher biomass following inoculation. The higher abundance of aldolase may increase the biomass by analogy to tobacco, where transgenes overexpressing plastid aldolase stimulated ribulose 1,5-bisphosphate regeneration and promoted CO_2_ fixation (Uematsu *et al.*, 2012). Furthermore, the transcript analysis indicated a differential ascorbate and glutathionine redox metabolism, which was supported by the finding of the differential abundance of two ascorbate peroxidases. The higher amount of tubulin and a cytoskeleton chaperone may indicate differences in the cytoskeleton after inoculation, but no evidence for transcriptomic changes in cytoskeleton genes was observed. In *Medicago*, mycorrhizal symbiosis increases the β-tubulin gene *MtTubb1* in infected root tissue, root nodules, the inner cortex, and the vasculature (Manthey *et al.*, 2004). In alfalfa nodule formation, regulation of tubulin is due to the microtubular cytoskeleton changes during symbiosis (Timmers *et al.*, 1998).

Phenylpropanoids and structural lignins are well known to be implicated in defence responses in plant–microbe interactions (Schenk *et al.*, 2000; Cheong *et al.*, 2002). In *Arabidopsis*, several members of the cinnamyl alcohol dehydrogenase gene family are induced in response to pathogenic *Pseudomonas* (Tronchet *et al.*, 2010). This parallels the observation for the *M. sinensis* cinnamyl alcohol dehydrogenase after inoculation with *H. frisingense*. Finally, little similarity in the differential proteomes is apparent in other endophyte-grass responses, such as the sugarcane response to the plant-growth-promoting bacterium *G. diazotrophicus* after 1 d (Lery *et al.*, 2011). This may partially be due to the fact that different sampling periods were used in the different studies, but as no general clues on plant responses in endophyte–plant interactions could be extracted from the transcriptomic responses, highly species-specific responses are suggested. Common effects include early jasmonate-related signalling in the entire plant, the local suppression of ethylene signalling to stimulate root growth, and changes in redox metabolism. Highly plant-specific interactions are also strongly supported by the fact that several studies identified genotype-specific responses within a single plant species.

Although not systematically analysed, we also noted unsuccessful inoculation attempts by *Herbaspirillum*. Low delivery rates are known to restrict the global potential of endophytes to colonize grasses in the field (Bhattacharjee *et al.*, 2008). Combinations of diverse bacteria in the inoculum (Fischer *et al.*, 2012) and/or mixtures of *Herbaspirillum* strains with other beneficial agents, such as humic acids (Canellas *et al.*, 2012), may provide a solution to improve the colonization efficiency in the field. As *H. frisingense* has beneficial effects on *Miscanthus* seedling growth and establishment, whereas the plant defence upon inoculation is minimal, *H. frisingense* may gain a more widespread use as a cheap biofertilizer.

## Supplementary data

Supplementary data are available at *JXB* online.

Supplementary Fig. S1. Exclusive detection of bacterial *H. frisingense* nitrogen-fixation regulator *nifA* in inoculated plants by PCR.

Supplementary Fig. S2. Localization of fluorescently labelled *H. frisingense* in the apoplast between leaf cells.

Supplementary Fig. S3. Increases in nutrient content (=dry biomass×nutrient concentration) of roots (A) and shoots (B) by *H. frisingense* inoculation.

Supplementary Fig. S4. Fertilization with ^15^N-enriched (10%) nutrient medium does not reveal nitrogen-fixation activities in *H. frisingense*-inoculated plants.

Supplementary Fig. S5. Transcriptome overview of *M. sinensis* grown for 3h with *H. frisingense*.

Supplementary Fig. S6. Transcriptome overview of *M. sinensis* grown for 3 weeks with *H. frisingense*.

Supplementary Fig. S7. Transcriptional categories affected by inoculation of *M. sinensis* with *H. frisingense*.

Supplementary Fig. S8. Influence of ethylene in growth promotion of *M. sinensis* by *H. frisingense*.

Supplementary Fig. S9. Effect of increasing ACC concentrations and *H. frisingense* on plant growth.

Supplementary Fig. S10. 2D gel electrophoresis of *M. sinensis* grown for 3 weeks without or with *H. frisingense*.

Supplementary Table S1. The primers used and predicted PCR fragment lengths.

Supplementary Materials **and** Methods. Details of the PCR conditions.

Supplementary Data

## Abbreviations:

ACC: 1-aminocyclopropane-1-carboxylate
IAA: indoleacetic acid
qRT-PCR: using quantitative real-time PCR.
