# Supplementary material for: Root ethylene signalling is involved in Miscanthus sinensis growth promotion by the bacterial endophyte Herbaspirillum frisingense GSF30T
Source: J Exp Bot. 2013 Sep 16;64(14):4603–15. doi: 10.1093/jxb/ert276 (PMC3808336; doi:10.1093/jxb/ert276)
Supplement: Supplementary Data [file supp_64_14_4603__index.html]

Root ethylene signalling is involved in Miscanthus sinensis growth promotion by the bacterial endophyte Herbaspirillum frisingense GSF30T — Root ethylene signalling is involved in Miscanthus sinensis growth promotion by the bacterial endophyte Herbaspirillum frisingense GSF30T — Supplementary Data 

# Root ethylene signalling is involved in *Miscanthus sinensis* growth promotion by the bacterial endophyte *Herbaspirillum frisingense* GSF30T

## Supplementary Data

Data files

**Files in this Data Supplement:**

- Supplementary Data - Supplementary Data
