# Supplementary material for: Root ethylene signalling is involved in Miscanthus sinensis growth promotion by the bacterial endophyte Herbaspirillum frisingense GSF30T
Source: J Exp Bot. 2013 Sep 16;64(14):4603–15. doi: 10.1093/jxb/ert276 (PMC3808336; doi:10.1093/jxb/ert276)
Supplement: Supplementary Data [file supp_ert276_jexbot103770_file001.pdf]

Supplementary information, including supporting figures:

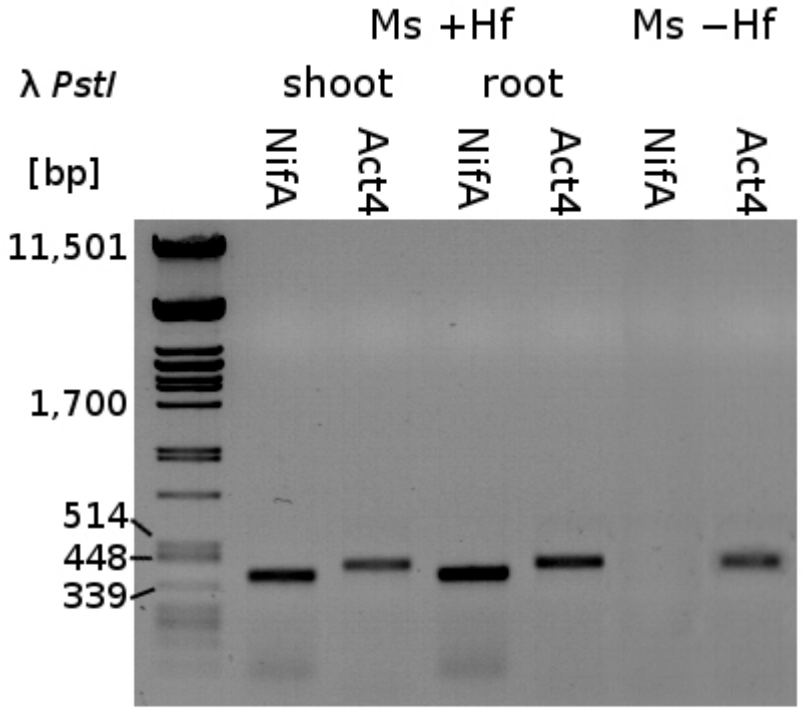

Suppl. Fig. S1: Exclusive detection of bacterial *H. frisingense* nitrogen fixation regulator *nifA* in inoculated plants by polymerase chain reaction. *H. frisingense* specific *NifA* gene was detected in roots and shoots of inoculated *Miscanthus sinensis*, but not in non-inoculated samples. *Act4* was amplified for quality verification of DNA-samples. *NifA*: nitrogen fixation regulator *NifA, Act4:* Actin-4.

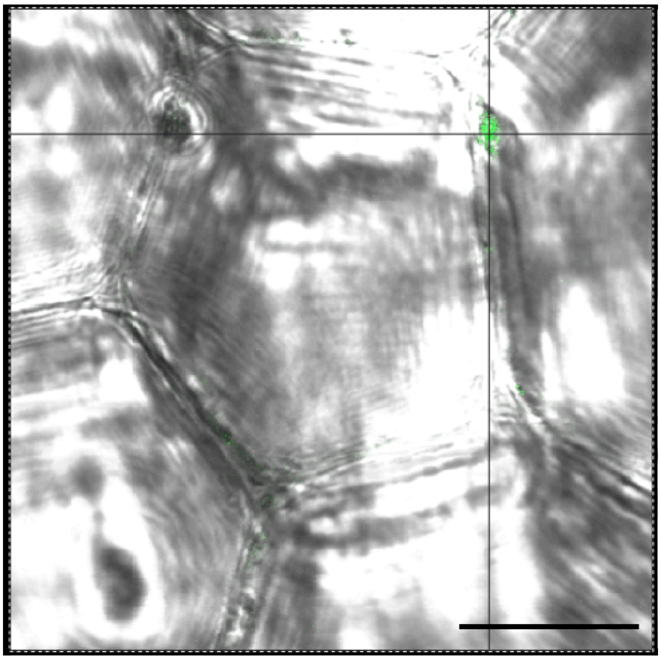

Suppl. Fig. S2: Localization of fluorescently labeled *Herbaspirillum frisingense* in the apoplast between leaf cells. Confocal picture of green fluorescent protein labeled *HSF30*^{T} strain in distinct spots in the shoot of *Miscanthus sinensis*. Scale bar 50 µm.

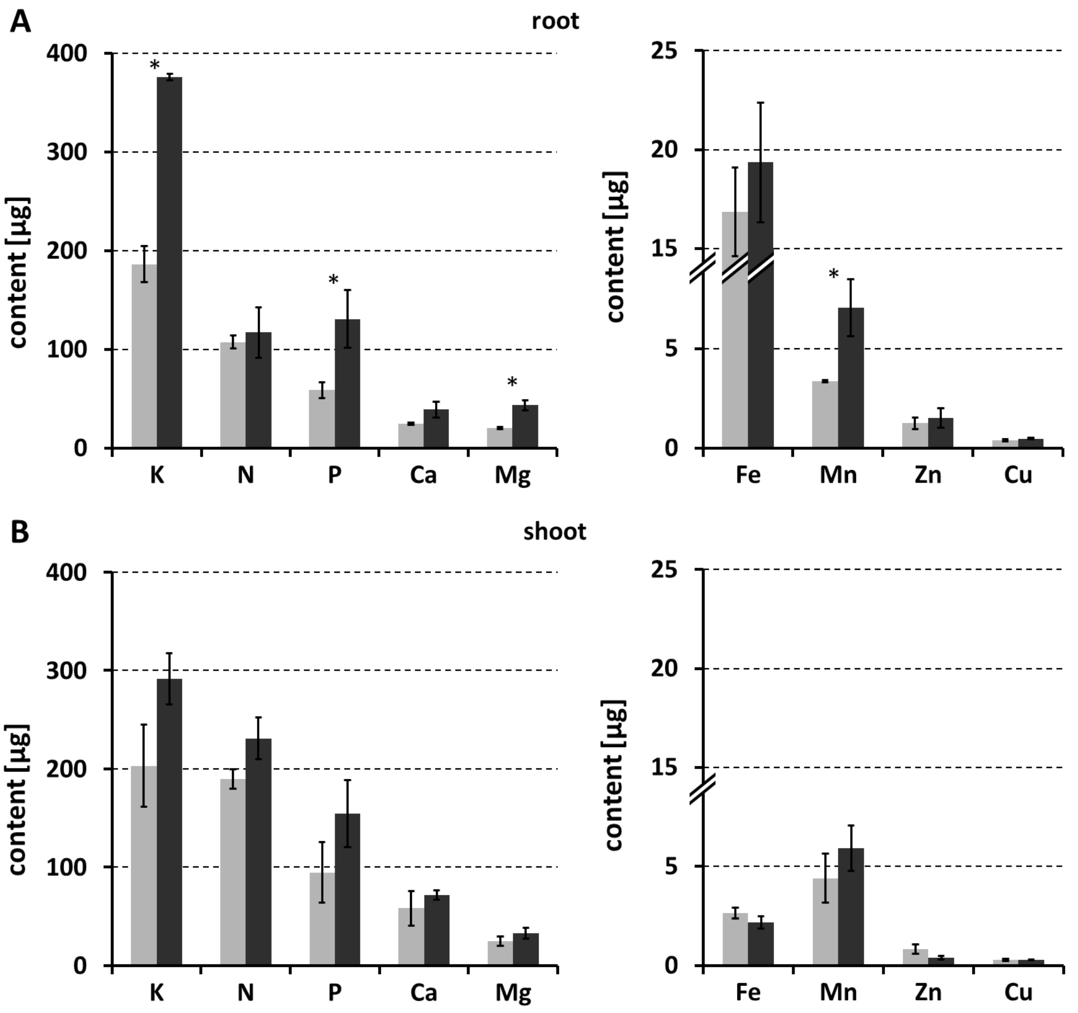

Suppl. Fig. S3: Increases in nutrient content (=dry biomass x nutrient concentration) of roots (A) and shoots (B) by *H. frisingense* inoculation. Macronutrient content (left) and micronutrient content (right) of control (grey bars) or inoculated (black bars) plant material. Significantly different content is given by an asterisk ($p<0.05$).

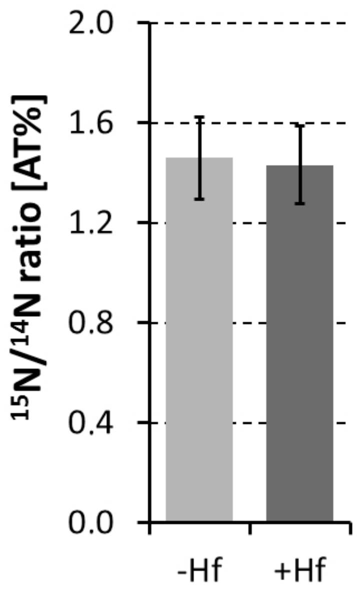

Suppl. Fig. S4: Fertilization with ^{15}N-enriched (10%) nutrient media did not reveal nitrogen fixation activities in *H. frisingense* inoculated plants. $^{15}N/^{14}N$ ratios in total plant material after 3 weeks without *H. frisingense* (left) and with *H. frisingense* (right).

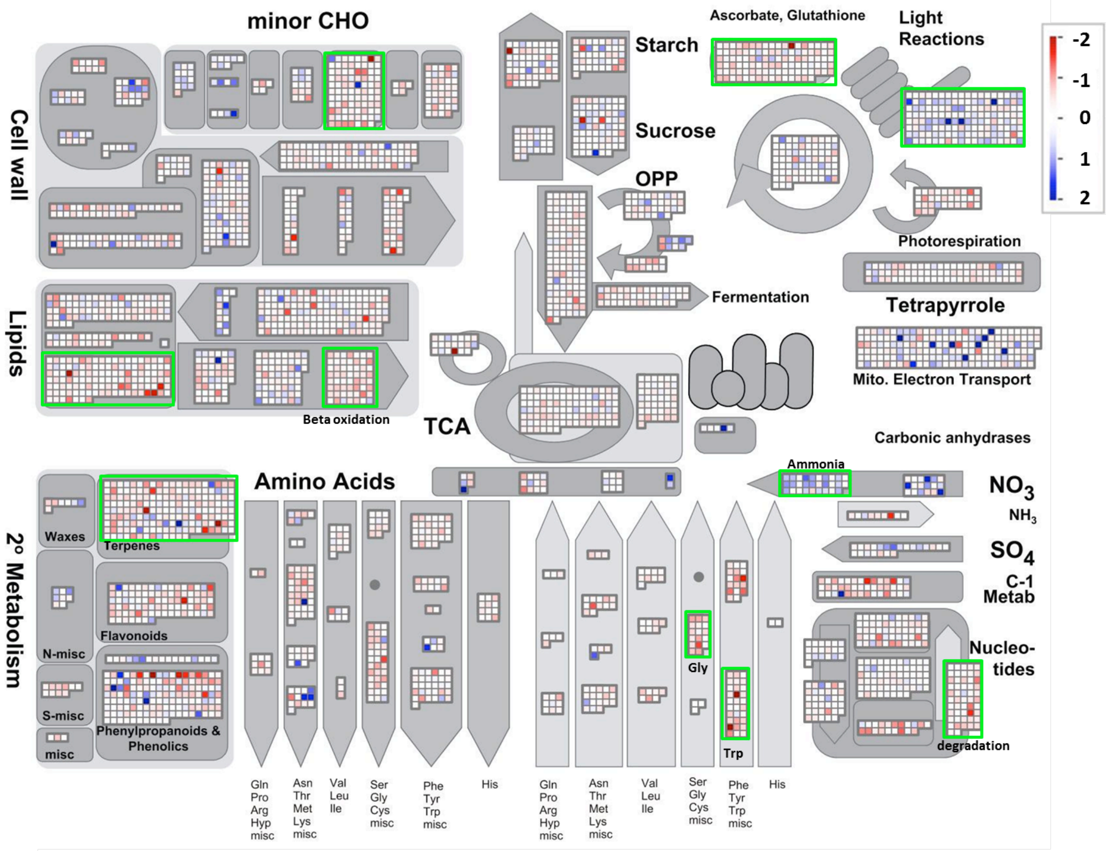

Suppl. Fig. S5: Transcriptome overview of *M. sinensis* grown 3 hours with *H. frisingense*. Higher expression in *M. sinensis* with *H. frisingense* is indicated by blue color and lower expression by red color. Logarithmic scale with white color is indicating similar expression level. Green squares around pathways indicates significant pathway regulation at *P*<0.001 by the Wilcoxon Rank Sum test, template from MAPMAN.

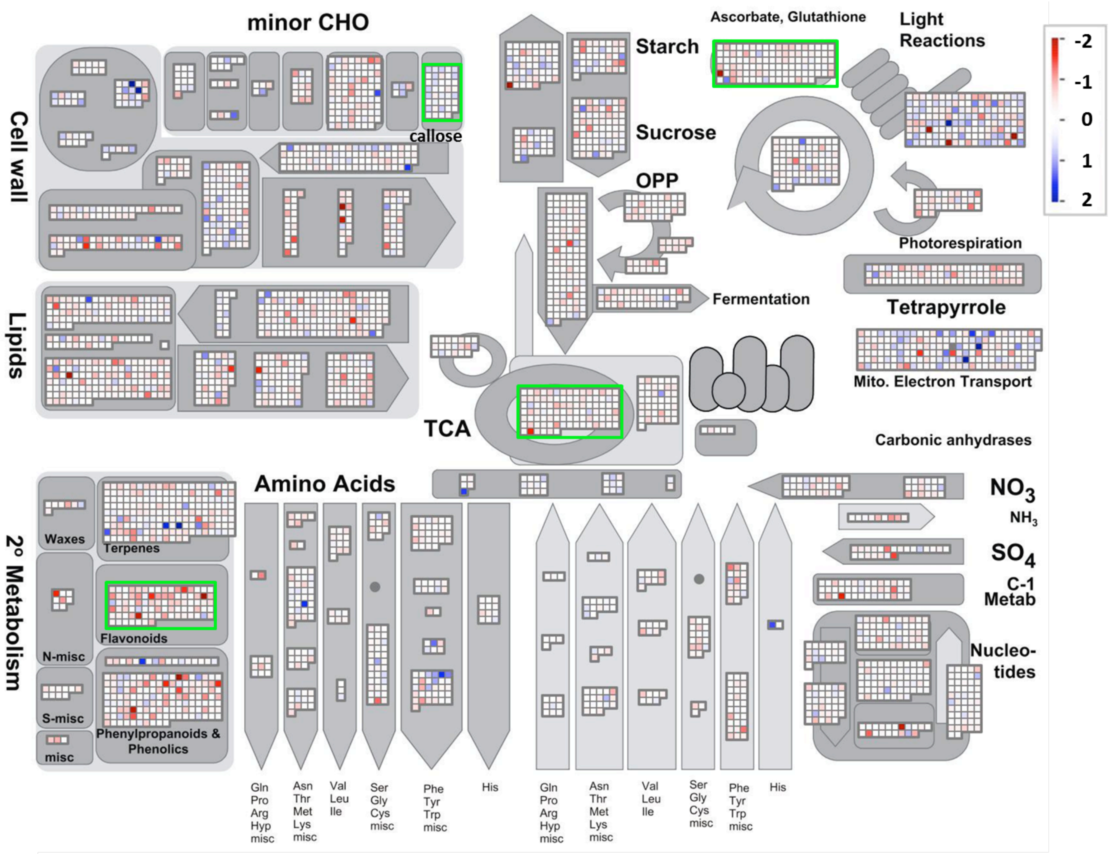

Suppl. Fig. S6: Transcriptome overview of *M. sinensis* grown 3 weeks with *H. frisingense*. Higher expression in *M. sinensis* with *H. frisingense* is indicated by blue color and lower expression by red color. Logarithmic scale with white color is indicating similar expression level. Green squares around pathways indicates significant pathway regulation at $P<0.003$ by the Wilcoxon Rank Sum test, template from MAPMAN.

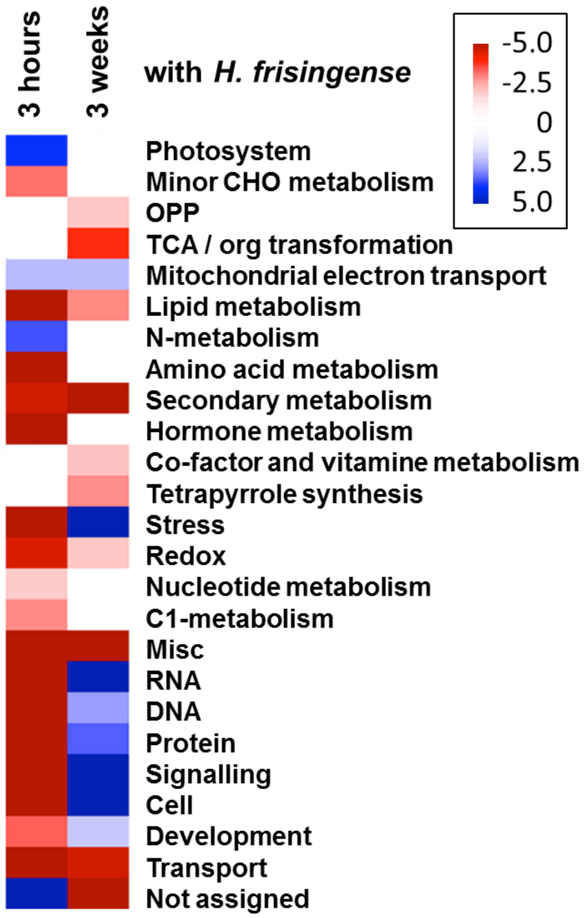

Suppl. Fig. S7: Transcriptional categories affected by inoculation of *M. sinensis* grown with *H. frisingense*. Significantly higher expression (z-score >1.96; ≈ $P<0.05$) is indicated by blue color, lower expression (z-score <-1.96; ≈ $P<0.05$) by red color (Bin-wise Wilcoxon test) and non-significant differences (z-score <1.96) are shown in white. *M. sinensis* grown 3 hours (left) or 3 weeks (right) with *H. frisingense* compared to non-inoculated plants.

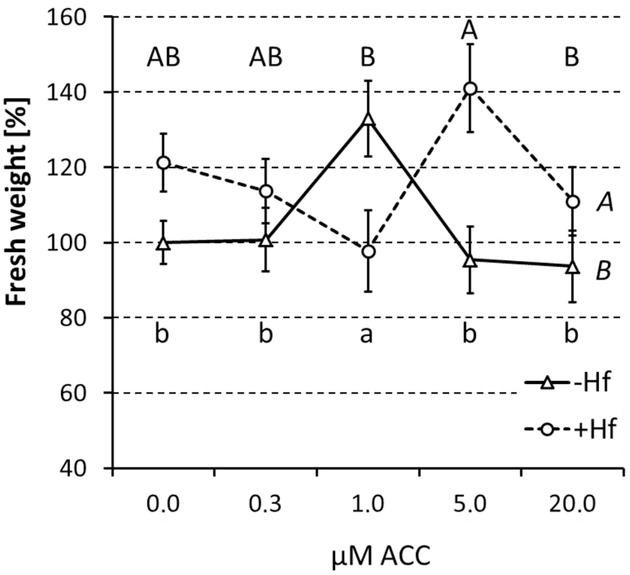

Suppl. Fig. S8: Influence of ethylene in growth promotion of *Miscanthus sinensis* by *H. frisingense.* Increased biomass (fresh weight in % to -Hf , ± SE, two replicates) with *H. frisingense* inoculation (12-14 plants) under low nitrogen with different concentrations of ACC (1-aminocyclopropane-1-carboxylic acid). Lower case letters indicate whether statistical differences were observed within the –Hf treatment, capital letters indicate statistical differences in the +Hf experiments. Capital italic letters indicate statistical differences between all –Hf and +Hf treatments (two-way ANOVA test, $P<0.05$).

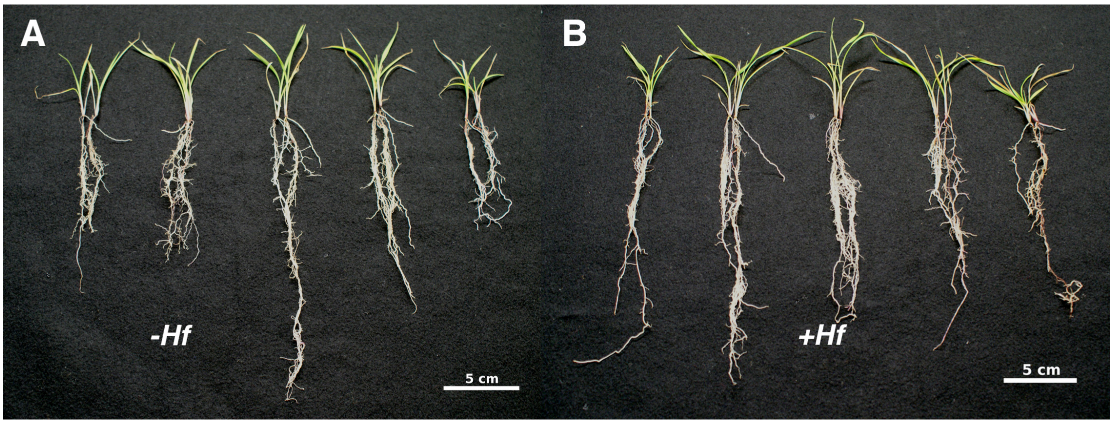

Suppl. Fig. S9: Effect of increasing ACC concentrations and *H. frisingense* on plant growth. Plants grown without *H. frisingense* (left) and with *H. frisingense* (right). Two plants for each treatment are shown. Increasing ACC concentrations in watering solution (in µM) from left to right: 0, 0.3, 1, 5, 20.

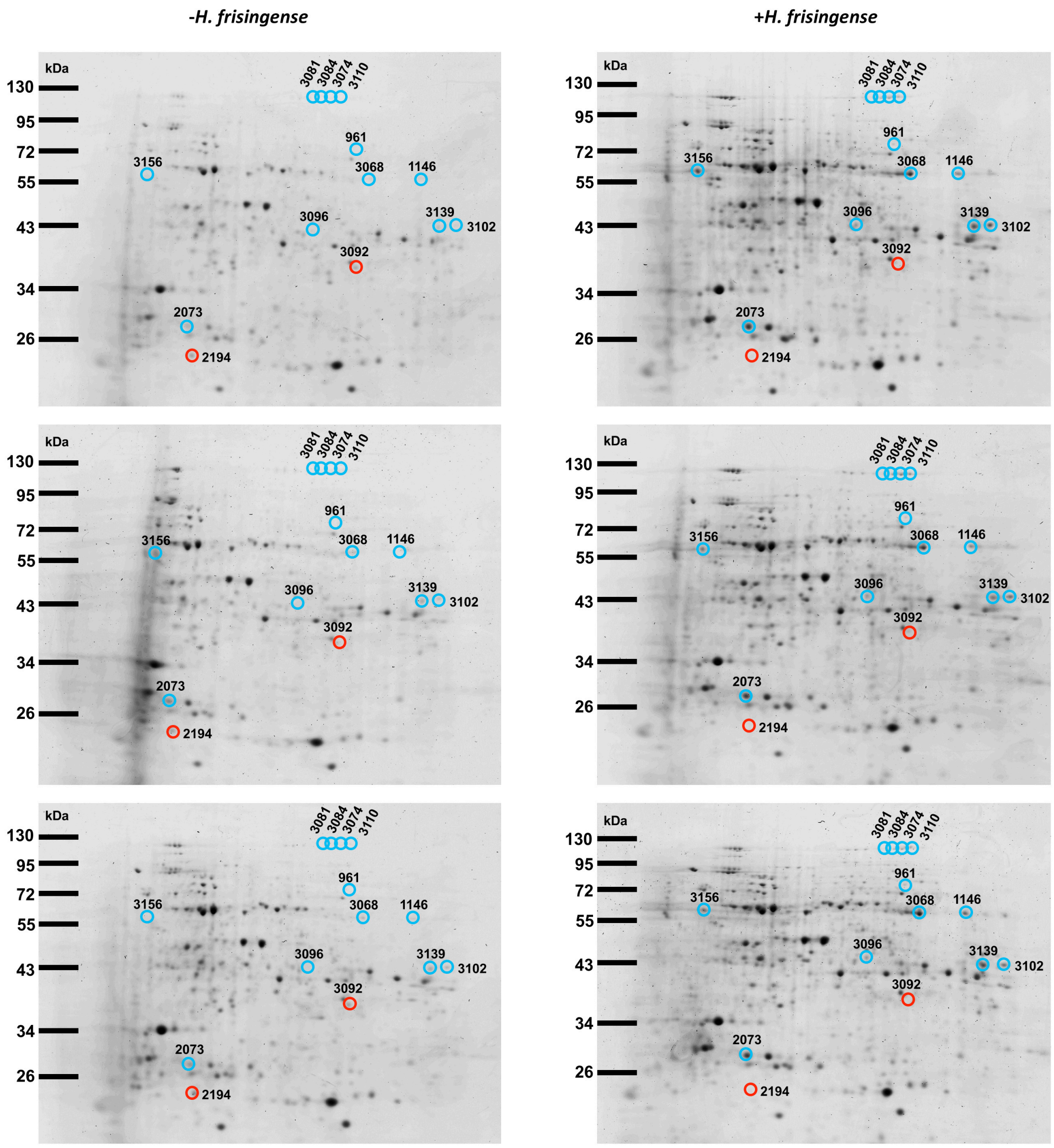

Suppl. Fig. S10: 2D gel electrophoresis of *M. sinensis* grown 3 weeks without (left) or with (right) *H. frisingense*. Protein spots significantly different compared to non-inoculated plants are indicated by circles. Blue color indicates significantly ($p<0.05$, >1.8-fold) more abundant protein spots after three weeks with *H. frisingense*, red color indicates lower abundance with *H. frisingense*. The underlying protein in a few differential protein spots

could not be unambiguously identified, in these cases the spots were neglected from further analysis.

Annotation	Primer sequence forward	reverse	Product length [bp]
PP2AA2	TGTTGAAGGTTGTGCTGCTC	GCGACCATATAACGAACACG	120
Actin 1	AGTTTCTGGGGAAGTTCGAC	GGTAACGAATCGGAGTCACTG	147
DnaJ Protein	TGTGGTTCATCCTCTGAAGG	ACCCTTACCATTGCACTTCG	106
BGAF-1	AAAGGTCGCGCAAAGGAG	CACACACTGGTTTGCTTTCC	93
BGAF-2	CCCGCAAAATAGAGTCAATG	TTGTAACTCAAAGAGCAGTGAGC	147
dirigent-like	GATGGGGACTTTTCCGATTAC	GCTCGCATCAACAGTGTACTTG	100
ARF1	ATTAGCCCACGGAGATGTTG	TGAGGGTGATGAATCTCCTG	91
ARF6	CGTTCTGTGAAGGTTGGTTG	AAAGGCAGTTGGGTACATCG	111
IAA18	TCTTCGACGGCAGATGAAAG	TAAGCAATCCCCTGAGCAAC	165
IAA20	CGTGAACGATGTTTCTGTCC	CGAAACCGTAACTTGACACG	146
EIN3	GTTGAAACCAGAACCGAACC	TGAGGGTTTCTTTGGACAGG	184
ETR1	GGCCCTTTCCAGAAGATTTG	CCGGAGATTTGCATTTGG	133
SCER1a	TCTCAATCCCTTTGGAGCTG	TTTGCCACTGTCAACACCAC	175
SCER1b	ATCAGCTCCAAAGGGATGAG	TGCAGCATCTCGATTAGTGC	153
SCERF1	AAGGCCAAGGTCAACTTCC	CCATAGTTTCAGGCTTCACG	175

Suppl. Table 1: Used primers and predicted PCR fragment length.

Supplementary material and methods

Polymerase chain reaction -- Plant samples were entire control plants or inoculated plants separated in shoot and root. DNA was extracted from 100mg frozen plant material, resuspended in 200 µl water and 1 µl was used in the PCR. The final PCR mix (50µl per sample) contained 1x Buffer, 200 nM each dNTP, 200 nM each Primer (Actin-4: Act4 fw TGCTGGTCGGGACCTCACGG, Act4 rv TGCTGCTTGGTGCAAGGGAAGT; NifA fw TCACTGCCACCAACCGCGAC, NifA rv CGGCACGACCACGACTGCTT) and 1 U Taq Polymerase. The lid-heated (104°C) PCR-Cycler (T3, Biometra) was programmed for 4 min 94°C initial denaturation, followed by 35 cycles 94°C 30 sec, 57°C 30 sec, 72°C 45 sec, and a final extension for 10 min at 72°C. An aliquot of the PCR was loaded on a Gel (1% Agarose in

TAE-Buffer), 8V/cm was applied 45 min and stained in Ethidium-Bromide.
